# Assessment of Sarcopenia Measures, Survival, and Disability in Older Adults Before and After Diagnosis With Cancer

**DOI:** 10.1001/jamanetworkopen.2020.4783

**Published:** 2020-05-12

**Authors:** Grant R. Williams, Yanjun Chen, Kelly M. Kenzik, Andrew McDonald, Shlomit S. Shachar, Heidi D. Klepin, Stephen Kritchevsky, Smita Bhatia

**Affiliations:** 1 Institute for Cancer Outcomes and Survivorship, University of Alabama at Birmingham; 2 Division of Oncology, Rambam Health Care Campus, Haifa, Israel; 3 Rappaport Faculty of Medicine, Technion-Israel Institute of Technology, Haifa, Israel; 4 Wake Forest School of Medicine, Wake Forest University, Winston-Salem, North Carolina

## Abstract

**Importance:**

Progressive loss of muscle mass and strength, known as sarcopenia, is a well-known phenomenon of aging; however, little is known about the trajectory of sarcopenia measures before and after cancer diagnosis and its contribution to subsequent disability.

**Objective:**

To examine the rate of decline of sarcopenia measures (ie, appendicular lean mass [ALM], muscle strength, and physical performance) in older adults with cancer both before and after the cancer diagnosis compared with the trajectory of a population without cancer, and secondarily to assess the association of sarcopenia measures with overall survival and major disability in patients with cancer.

**Design, Setting, and Participants:**

This matched cohort study included participants from the Health, Aging, and Body Composition (Health ABC) study, which included 3075 community-dwelling older adults aged 70 to 79 years recruited from a random sample of white Medicare beneficiaries and all eligible black residents in and around Pittsburgh, Pennsylvania, and Memphis, Tennessee, beginning in January 1997 and observed for 17 years until December 2013. Data were analyzed from May 2018 to February 2020.

**Exposures:**

The development of an adjudicated cancer diagnosis confirmed with pathology or cytology reports during the first 7 years of follow-up.

**Main Outcomes and Measures:**

Annual assessments of ALM, hand grip strength, and gait speed were the primary outcome measures. Linear mixed-effect models were used to compare the change in ALM, hand grip strength, and gait speed between individuals who developed cancer and those who did not, adjusted for multiple comparisons (*P* < .01). Multivariable Cox regression was used to examine the association of sarcopenia measures with overall survival and major disability from date of cancer diagnosis.

**Results:**

Of the 3075 included patients, 1491 (48.5%) were male, 1281 (41.7%) were black, and the mean (SD) age was 74.1 (2.9) years. A total of 515 patients (16.7%) developed cancer within the first 7 years of the study. The most common cancers were prostate (117 [23.2%]), colorectal (63 [12.5%]), lung (61 [12.1%]), and breast (61 [12.1%]) cancer, and 165 patients (32.0%) were diagnosed as having metastatic disease. Compared with controls without cancer, patients with a cancer diagnosis had a steeper decline in gait speed (β = −0.02; 95% CI, −0.03 to −0.01; *P* < .001) but not ALM (β = −0.02; 95% CI, −0.07 to 0.04; *P* = .49) or hand grip strength (β = −0.21; 95% CI, −0.43 to 0; *P* = .05) prior to cancer diagnosis. After cancer diagnosis, there was a decline in ALM (β = −0.14; 95% CI, −0.23 to −0.05; *P* < .001) but not hand grip strength (β = −0.02; 95% CI, −0.37 to 0.33; *P* = .92) or gait speed (β = 0; 95% CI, −0.01 to 0.02; *P* = .51). Declines in ALM after a cancer diagnosis were most striking in patients with metastases (β = −0.32; 95% CI, −0.53 to −0.10; *P* = .003). Slow gait speed was associated with a 44% increase in mortality (hazard ratio, 1.44; 95% CI, 1.05 to 1.98; *P* = .02) and a 70% increase in disability (hazard ratio, 1.70; 95% CI, 1.08 to 2.68; *P* = .02) but not low ALM or hand grip strength.

**Conclusions and Relevance:**

Accelerated losses in differing sarcopenia measures exist both prior to and after a cancer diagnosis and may present opportunities for targeted interventions to improve outcomes.

## Key Points

**Question:**

What is the association of a cancer diagnosis with measures of sarcopenia?

**Findings:**

In this matched cohort study of 515 older patients who developed cancer from the Health, Aging, and Body Composition (Health ABC) study, a steeper decline in gait speed prior to a cancer diagnosis and accelerated declines in appendicular lean mass after a cancer diagnosis were found, which were most striking in patients with metastatic disease.

**Meaning:**

Accelerated losses in differing sarcopenia measures exist both prior to and after a cancer diagnosis and may present opportunities for targeted interventions to improve outcomes.

## Teaser

This matched cohort study examines the rate of decline of sarcopenia measures in older adults with cancer both before and after the cancer diagnosis.

## Introduction

Age-related loss in muscle mass and strength, known as sarcopenia, is detected as early as the 4th decade of life and shows a linear age-related decline.^1,2^ Age-related sarcopenia may be further complicated by the development of cancer and related cancer treatments.^3^ Low skeletal muscle mass is highly prevalent in older adults with cancer and strongly associated with a higher risk of adverse events, such as chemotherapy-related toxic effects, surgical complications, and poorer overall survival.^4,5,6,7^ Cancer cachexia is a multifactorial syndrome caused by an elevated inflammatory response combined with reduced food intake that may contribute to additional muscle loss in patients with cancer.^8,9,10^ However, the contribution of cancer to the trajectory of sarcopenia both preceding and after a cancer diagnosis remains unknown. Furthermore, the association of sarcopenia at cancer diagnosis with subsequent disability in older adults with cancer also remains largely unknown.^3^

To fill these gaps, we examined the rate of decline of sarcopenia indices (ie, appendicular lean mass [ALM], muscle strength, and physical performance) in older adults with cancer before and after cancer diagnosis compared with the trajectory of a population without cancer. We also assessed the association of these sarcopenia indices with overall survival and major disability in patients with cancer.

## Methods

### Sample

The Health, Aging, and Body Composition (Health ABC) study^11^ was a prospective longitudinal observational study designed to assess the association of body composition with physical function and disability in older adults. The study enrolled 3075 well-functioning community-dwelling older adults aged 70 to 79 years at enrollment from a random sample of white Medicare beneficiaries and all eligible black residents in designated zip code areas in and around Pittsburgh, Pennsylvania, and Memphis, Tennessee, between March 1997 and July 1998. At enrollment, participants reported no difficulty performing activities of daily living, walking 0.25 miles, or climbing 10 steps without resting. Each local institutional review board approved the protocol, and all participants provided written informed consent. The current secondary analyses were considered exempt under category 4 of federal regulations. Follow-up clinical examinations or home-based interviews were performed annually, and short telephone interviews were performed between each annual interview at 6 months. A report of a new cancer diagnosis during follow-up assessments prompted an adjudication protocol with procurement of pathology and cytology reports as well as supportive radiologic and laboratory data (nonmelanoma skin cancer was counted as control). Date of diagnosis, primary cancer site, and presence of metastases were documented. The study period includes cancer reported and adjudicated through December 2004 and follow-up for mortality and major disability through December 2013. This study followed the Strengthening the Reporting of Observational Studies in Epidemiology (STROBE) reporting guideline.^12^

### Sarcopenia Indices

Appendicular lean mass (in kilograms) was assessed by summing lean tissue (fan-beam dual-energy radiography absorptiometry 4500A version 8.20a [Hologic]) across all extremities. Low ALM was defined as less than 19.75 kg for men and less than 15.02 kg for women.^13^ Hand grip strength was assessed using the JAMAR hydraulic handheld dynamometer (MESM) and computed as an average from 2 trials of each hand. A hand grip strength less than 26 kg for men and less than 16 kg for women was used to define low hand grip strength.^13^ Gait speed was evaluated over a 20-m course and examined as a continuous variable, with a value of 0.8 m/s or less considered abnormal.^14,15^

### Covariates

Demographic characteristics included age, sex, race/ethnicity, education, and study site. Cancer-related information included cancer site and cancer stage (limited vs metastatic).

### Outcomes

Sarcopenia indices constituted the primary outcome variables. Secondary outcomes included overall survival and major disability after cancer diagnosis; these analyses were restricted to the cancer cohort. Major disability was defined as requiring a cane or walker for ambulation, inability to walk 0.25 miles or climb 10 steps or more, or requiring assistance with activities of daily living, including transferring, dressing, or bathing.

### Statistical Analyses

Given sex differences between those with and without cancer, we selected a 2:1 match of participants without cancer to participants with cancer based on sex, race/ethnicity, education, and age at enrollment. In addition, we separately performed a 5:1 match for propensity scores to develop controls without cancer for patients with prostate, colorectal, lung, breast, and other cancers, as well as those with metastatic disease. Controls without cancer were assigned an age at pseudodiagnosis, based on average age at diagnosis for the patients with cancer. Greedy matching technique was used with a caliper width equal to 0.2 in the matching.^16^ Using the matched sets, we used linear mixed-effect models with patient-level random intercepts to compare the slopes in each of the 3 sarcopenia indices (ALM, hand grip strength, and gait speed) between individuals who did and did not develop cancer, further adjusting for age at diagnosis, race/ethnicity, sex, education, enrollment site, and longitudinal comorbidities, including diabetes, cardiovascular disease, and arthritis at each measurement. Given concern for multiple comparisons, we chose a stricter significance threshold of a 2-tailed *P* value less than .01. The slopes were examined for the available period before cancer diagnosis (censoring at 1 month before cancer diagnosis) and for the period from cancer diagnosis to the last evaluation in patients with cancer. For determining the association between overall survival and sarcopenia measures, we used multivariable Cox regression; the at-risk period included time from cancer diagnosis to death or last contact (if alive). For determining the association between major disability and sarcopenia measures, we used multivariable proportional subdistribution hazards regression (Fine-Gray method) using death as a competing risk. Dichotomized thresholds of impairment for each sarcopenia measure were treated as time-varying covariates. Missing data (minimal) were treated as missing at random with no imputations; all analyses used available data. SAS statistical software version 9.4 (SAS Institute) was used for analyses.

## Results

Of the 3075 included patients from the Health ABC study, 1491 (48.5%) were male, 1281 (41.7%) were black, and the mean (SD) age was 74.1 (2.9) years (Table 1). A total of 515 patients (16.7%) were diagnosed as having cancer during the first 7 years of the study. Participants were observed for a median (range) of 3.2 (0 to 6.9) years prior to cancer diagnosis and 0.5 (0 to 5.3) years after cancer diagnosis. The cancer subcohort was observed for a median (range) of 4.2 (0 to 17) years and 0.5 (0 to 17) years after cancer diagnosis for mortality and major disability analyses, respectively. Most common cancers included prostate (117 [23.2%]), colorectal (63 [12.5%]), lung (61 [12.1%]), and breast (61 [12.1%]) cancer. Metastatic disease was present in 165 patients (32.0%) at cancer diagnosis. Baseline values for ALM, hand grip strength, and gait speed were comparable for the cancer and noncancer cohorts (Table 2).

**Table 1.** Patient Characteristics^a^

Characteristic	No. (%)		
	Total sample (N = 3075)	Patients with cancer (n = 515)	Matched controls (n = 1030)
Age, mean (SD), y^a^	74.1 (2.9)	74.4 (2.9)	74.4 (2.9)
Male	1491 (48.5)	323 (62.7)	646 (62.7)
Race			
White	1794 (58.3)	279 (54.2)	565 (54.9)
Black	1281 (41.7)	236 (45.8)	465 (45.2)
Site			
Pittsburgh, Pennsylvania	1548 (50.3)	264 (51.7)	507 (49.2)
Memphis, Tennessee	1527 (49.7)	251 (48.7)	523 (50.8)
Incident cancer site of origin			
Prostate	NA	117 (23.2)	NA
Colorectal	NA	63 (12.5)	NA
Lung	NA	61 (12.1)	NA
Breast	NA	61 (12.1)	NA
Other	NA	213 (42.2)	NA
Cancer stage			
Limited	NA	293 (56.9)	NA
Metastatic	NA	165 (32.0)	NA
Unknown	NA	57 (11.1)	NA

Abbreviation: NA, not applicable.

^a^ Variables presented are baseline values at enrollment in the study.

**Table 2.** Sarcopenia Indices and Long-term Outcomes

Measure	Mean (SD)		
	Total sample (N = 3075)	Patients with cancer (n = 515)	Matched controls (n = 1030)
**Sarcopenia indices**			
Appendicular lean mass, kg			
Female participants	16.6 (3.2)	16.6 (3.0)	16.4 (3.2)
Male participants	23.9 (3.6)	24.1 (3.6)	23.8 (3.6)
Hand grip strength, kg			
Female participants	22.5 (5.5)	21.9 (5.4)	22.6 (5.6)
Male participants	37.3 (8.2)	38.6 (8.4)	36.8 (8.0)
Gait speed, m/s	1.33 (0.26)	1.35 (0.25)	1.34 (0.25)
**Long-term outcomes**			
Died, No. (%)^a^	1991 (64.8)	413 (80.2)	676 (65.6)
Major disability, No. (%)^a^	1107 (36.0)	283 (55.0)	665 (64.6)

^a^ Denotes significant difference between patients with cancer and matched controls.

Among individuals who developed cancer during the study period, we found significantly steeper declines in gait speed (β = −0.02; 95% CI, −0.03 to −0.01; *P* < .001) but not ALM (β = −0.02; 95% CI, −0.07 to 0.04; *P* = .49) or hand grip strength (β = −0.21; 95% CI, −0.43 to 0; *P* = .05) prior to cancer diagnosis compared with controls without cancer (Figure). Declines in gait speed before a cancer diagnosis were observed in patients with prostate cancer (β = −0.02; 95% CI, −0.04 to −0.01; *P* = .002), metastatic disease (β = −0.03; 95% CI, −0.04 to −0.02; *P* < .001), and other cancers (β = −0.03; 95% CI, −0.04 to −0.01; *P* < .001) (Table 3). No significant declines in hand grip strength before a cancer diagnosis were found that reached the predefined statistical significance threshold. After cancer diagnosis, there was a significant decline in ALM (β = −0.14; 95% CI, −0.23 to −0.05; *P* < .001) but not hand grip strength (β = −0.02; 95% CI, −0.37 to 0.33; *P* = .92) or gait speed (β = 0; 95% CI, −0.01 to 0.02; *P* = .51). Declines in ALM after cancer diagnosis was most striking in patients with metastatic disease (β = −0.32; 95% CI, −0.53 to −0.10; *P* < .001) (Figure).

**Figure.**
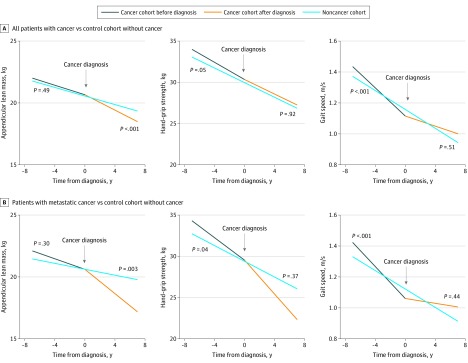
Comparison of Sarcopenia Indices In Patients Before and After Cancer Diagnosis With Controls Without Cancer

**Table 3.** Difference in Sarcopenia Indices Between the Participants With Cancer and Matched Controls Without Cancer Before and After Cancer Diagnosis^a^

Index	Appendicular lean mass		Hand grip strength		Gait speed	
	Estimate, β (95% CI)	*P* value	Estimate, β (95% CI)	*P* value	Estimate, β (95% CI)	*P* value
All cancer vs none						
Before diagnosis	−0.02 (−0.07 to 0.04)	.49	−0.21 (−0.43 to 0)	.05	−0.02 (−0.03 to −0.01)	<.001
After diagnosis	−0.14 (−0.23 to −0.05)	<.001	−0.02 (−0.37 to 0.33)	.92	0 (−0.01 to 0.02)	.51
Prostate cancer vs none						
Before diagnosis	0.03 (−0.09 to 0.16)	.60	−0.58 (−1.09 to −0.06)	.03	−0.02 (−0.04 to −0.01)	.002
After diagnosis	−0.12 (−0.27 to 0.03)	.13	0.07 (−0.57 to 0.71)	.83	0 (−0.02 to 0.02)	.87
Colorectal vs none						
Before diagnosis	−0.09 (−0.23 to 0.06)	.25	−0.65 (−1.24 to −0.05)	.03	−0.01 (−0.03 to 0.01)	.50
After diagnosis	−0.15 (−0.40 to 0.09)	.22	−0.06 (−1.08 to 0.95)	.90	0.01 (−0.03 to 0.05)	.63
Lung cancer vs none						
Before diagnosis	0 (−0.16 to 0.16)	.99	−0.46 (−1.07 to 0.15)	.14	−0.02 (−0.04 to 0)	.04
After diagnosis	−0.23 (−0.65 to 0.20)	.30	−0.30 (−2.02 to 1.42)	.73	0.03 (−0.03 to 0.08)	.33
Breast cancer vs none						
Before diagnosis	−0.01 (−0.14 to 0.11)	.82	0.07 (−0.35 to 0.48)	.75	−0.02 (−0.04 to 0)	.06
After diagnosis	−0.11 (−0.31 to 0.09)	.27	−0.02 (−0.72 to 0.68)	.96	0.01 (−0.02 to 0.04)	.49
Other cancer vs none						
Before diagnosis	−0.04 (−0.13 to 0.04)	.28	0.10 (−0.21 to 0.42)	.51	−0.03 (−0.04 to −0.01)	<.001
After diagnosis	−0.12 (−0.27 to 0.02)	.10	−0.08 (−0.67 to 0.50)	.78	0 (−0.02 to 0.02)	.85
Metastatic vs none						
Before diagnosis	−0.05 (−0.14 to 0.04)	.30	−0.39 (−0.76 to −0.02)	.04	−0.03 (−0.04 to −0.02)	<.001
After diagnosis	−0.32 (−0.53 to −0.10)	.003	−0.39 (−1.24 to 0.46)	.37	0.01 (−0.02 to 0.04)	.44

^a^ Based on linear mixed-effect model using matched set, further adjusting for race/ethnicity, sex, age at enrollment, education, and longitudinal measurements of diabetes, cardiovascular disease, and arthritis. Given concern for multiple comparisons, we chose a stricter significance threshold of *P* less than .01.

Slow gait speed was associated with a 44% increase in mortality (hazard ratio [HR], 1.44; 95% CI, 1.05 to 1.98; *P* = .02) and a 70% increase in disability (HR, 1.70; 95% CI, 1.08 to 2.68; *P* = .02). Low ALM and low hand grip strength were not associated with increased mortality (ALM: HR, 1.02; 95% CI, 0.80 to 1.30; *P* = .90; hand grip strength: HR, 1.18; 95% CI, 0.91 to 1.54; *P* = .22) or increased disability (ALM: HR, 0.91; 95% CI, 0.71 to 1.17; *P* = .47; hand grip strength: HR, 1.29; 95% CI, 0.98 to 1.69; *P* = .07).

## Discussion

Accelerated losses in differing sarcopenia indices were found among older adults before and after cancer diagnosis compared with a comparison group without cancer. Decline in gait speed was observed prior to cancer diagnosis, and accelerated decline in ALM was seen after cancer diagnosis, particularly in those with metastatic disease. Only low gait speed was associated with increased mortality and disability. The Health ABC study offered a rare opportunity to examine changes in sarcopenia indices before and after cancer diagnosis and provides one of the first comparisons of cancer-related declines in sarcopenia measures compared with a population without cancer.

After cancer diagnosis, there was an accelerated loss of ALM compared with normal declines related to aging. In a 2018 review,^17^ several studies reported similar declines in muscle mass during chemotherapy. In a pilot study by Stene et al^18^ of patients with advanced lung cancer receiving palliative chemotherapy, they found a 4.6-cm^2^ loss in skeletal muscle cross-sectional area, corresponding to a 1.4-kg loss of whole-body muscle mass. Similarly, patients with cholangiocarcinoma undergoing chemotherapy lost 1.2 kg after 100 days of therapy.^19^ In another small study by Blauwhoff-Buskermolen et al^20^ of patients undergoing systemic chemotherapy for metastatic colorectal cancer, muscle area declined by 6.1% after 3 months of treatment. Notably, in 2 of these studies,^18,20^ losses of muscle mass during chemotherapy were associated with inferior survival. Although these studies have suggested declines in muscle mass during chemotherapy, to our knowledge, our study is the first to provide a comparison population of participants without cancer with normal age-related sarcopenia losses. Furthermore, as our study involved many patients with early-stage disease, it suggests that worsening sarcopenia could be an underrecognized survivorship issue in oncology and warrants further examination.

Accelerated declines in gait speed leading up to cancer diagnosis may be caused by symptoms, hospitalizations, and/or surgery associated with a cancer diagnosis. Although gait speed is a relatively simple measurement to obtain, walking is a complex task involving multiple systems that must work in concert.^21^ Based on the Figure, it appears that the losses in gait speed preceding cancer diagnosis are largely recovered after cancer diagnosis, potentially suggesting a more acute decline, as would be anticipated from a hospitalization. However, it was surprising to find significant declines in gait speed before cancer diagnosis specifically within individuals with prostate cancers, as many of these individuals likely had asymptomatic and localized disease. The association of gait speed with mortality and disability is consistent with recent results in oncology and again highlights the importance of gait speed as a potential marker of frailty and as a critical tool in risk prediction in older adults with cancer.^21,22,23^

The lack of association of low ALM with mortality is in contrast with previous reports in oncology; however, the vast majority of previous literature within cancer have relied on single-slice computed tomography for measuring body composition and did not use dual-energy radiography absorptiometry to assess muscle mass.^24^ Additionally, our findings suggest potential discrepancies in the accelerated losses in sarcopenia measures. Although these measures all represent different facets of sarcopenia, the relationship between muscle mass, muscle strength, and physical performance is not linear, and prior research has shown only modest correlation.^25,26,27^ Notably, in a study of older adults with cancer, there was no association between the skeletal muscle mass and physical performance impairments.^28^ Muscle strength and physical performance do not depend solely on muscle mass, and many other factors complicate this relationship, including muscle composition.^29^

Although the terms *sarcopenia* and *cachexia* are often used interchangeably within the context of cancer, these represent distinct entities.^30^ Similar to sarcopenia, cachexia is associated with a decrease in muscle mass, muscle strength, and function and is also associated with losses in body weight as well as an increase in basal metabolic rate and markers of inflammation.^8,10,31^ Within the older adult population with cancer, depending on the cancer type and stage, both of these entities are likely present in varying degrees. Our goal in this study was to isolate the contribution of a cancer diagnosis to declines in indices of sarcopenia compared with a comparison group without cancer, but we are unable to delineate whether our findings are a result of sarcopenia or cancer cachexia. Regardless of the underlying cause, our results suggest worsening of gait speed before and declines in ALM after diagnosis, both of which are clinically important measures that have been associated with adverse outcomes in the older adult population.^21,22,32^ Further studies are warranted to investigate the causes of muscle loss and decline in gait speed in this population to inform appropriately targeted interventions.

### Limitations

This study should be considered in the context of its limitations. The older participants within the Health ABC study were recruited from only 2 US cities (Pittsburgh and Memphis) and were required to be well functioning at baseline, thus limiting the generalizability of our findings. Although the Health ABC study identified and confirmed new cancer diagnoses, no cancer treatment information was obtained, and we were unable to examine the association of specific treatments with trajectories of specific sarcopenia indices. Although the Health ABC study was a large study including more than 3000 participants, only 16.7% of participants developed cancer, and many of the subgroup analyses within specific cancer types were likely underpowered. Furthermore, the development of sarcopenia is often multifactorial and a multitude of events and conditions can influence this process, and we were not able to account for all events (eg, hospitalizations, weight loss, comorbid conditions) that may have contributed to the decline.

## Conclusions

These limitations notwithstanding, this study affords a rare glimpse of sarcopenia indices both before and after cancer diagnosis compared with a population without cancer. Accelerated loses in gait speed prior to cancer diagnosis and accelerated loses in ALM after cancer diagnosis suggest that a cancer diagnosis does affect age-related losses in some sarcopenia indices. However, many unanswered questions remain. Are these losses the result of cancer cachexia or cancer treatment? Do these accelerated losses represent a true increase in the rate of age-related decline or rather a phase-shift due to acute effects of cancer and cancer treatment? What are the best interventions to mitigate the decline in sarcopenia indices? Further work is needed to provide a deeper understanding of the effect of cancer and specific cancer treatments on the decline in sarcopenia indices in cancer survivors to inform effective interventions to combat sarcopenia in this growing population.

## References

[1] Metter EJ, Conwit R, Tobin J, Fozard JL Age-associated loss of power and strength in the upper extremities in women and men. J Gerontol A Biol Sci Med Sci. 1997;52(5):B267-B276. doi:10.1093/gerona/52A.5.B267 9310077

[2] Rolland Y, Czerwinski S, Abellan Van Kan G, Sarcopenia: its assessment, etiology, pathogenesis, consequences and future perspectives. J Nutr Health Aging. 2008;12(7):433-450. doi:10.1007/BF02982704 18615225 PMC3988678

[3] Williams GR, Rier HN, McDonald A, Shachar SS Sarcopenia & aging in cancer. J Geriatr Oncol. 2019;10(3):374-377. 30343999 10.1016/j.jgo.2018.10.009 PMC6472991

[4] Kazemi-Bajestani SM, Mazurak VC, Baracos V Computed tomography-defined muscle and fat wasting are associated with cancer clinical outcomes. Semin Cell Dev Biol. 2016;54:2-10. 26343952 10.1016/j.semcdb.2015.09.001

[5] Rier HN, Jager A, Sleijfer S, Maier AB, Levin MD The prevalence and prognostic value of low muscle mass in cancer patients: a review of the literature. Oncologist. 2016;21(11):1396-1409. doi:10.1634/theoncologist.2016-0066 27412391 PMC5189631

[6] Shachar SS, Deal AM, Weinberg M, Skeletal muscle measures as predictors of toxicity, hospitalization, and survival in patients with metastatic breast cancer receiving taxane-based chemotherapy. Clin Cancer Res. 2017;23(3):658-665. 27489287 10.1158/1078-0432.CCR-16-0940 PMC5290138

[7] Brown JC, Cespedes Feliciano EM, Caan BJ The evolution of body composition in oncology-epidemiology, clinical trials, and the future of patient care: facts and numbers. J Cachexia Sarcopenia Muscle. 2018;9(7):1200-1208. doi:10.1002/jcsm.12379 30637983 PMC6351674

[8] Fearon K, Arends J, Baracos V Understanding the mechanisms and treatment options in cancer cachexia. Nat Rev Clin Oncol. 2013;10(2):90-99. doi:10.1038/nrclinonc.2012.209 23207794

[9] Evans WJ, Morley JE, Argilés J, Cachexia: a new definition. Clin Nutr. 2008;27(6):793-799. doi:10.1016/j.clnu.2008.06.013 18718696

[10] Evans WJ Skeletal muscle loss: cachexia, sarcopenia, and inactivity. Am J Clin Nutr. 2010;91(4):1123S-1127S. doi:10.3945/ajcn.2010.28608A 20164314

[11] Introducing the Health ABC Study: the dynamics of health, aging, and body composition. Accessed February 25, 2020. https://healthabc.nia.nih.gov/

[12] Vandenbroucke JP, von Elm E, Altman DG, ; STROBE Initiative Strengthening the Reporting of Observational Studies in Epidemiology (STROBE): explanation and elaboration. Epidemiology. 2007;18(6):805-835. doi:10.1097/EDE.0b013e3181577511 18049195

[13] Studenski SA, Peters KW, Alley DE, The FNIH Sarcopenia Project: rationale, study description, conference recommendations, and final estimates. J Gerontol A Biol Sci Med Sci. 2014;69(5):547-558. doi:10.1093/gerona/glu010 24737557 PMC3991146

[14] Cruz-Jentoft AJ, Baeyens JP, Bauer JM, ; European Working Group on Sarcopenia in Older People Sarcopenia: European consensus on definition and diagnosis: report of the European Working Group on Sarcopenia in Older People. Age Ageing. 2010;39(4):412-423. doi:10.1093/ageing/afq034 20392703 PMC2886201

[15] Studenski S, Perera S, Patel K, Gait speed and survival in older adults. JAMA. 2011;305(1):50-58. doi:10.1001/jama.2010.1923 21205966 PMC3080184

[16] Austin PC A comparison of 12 algorithms for matching on the propensity score. Stat Med. 2014;33(6):1057-1069. doi:10.1002/sim.6004 24123228 PMC4285163

[17] Hopkins JJ, Sawyer MB Interactions of lean soft-tissue and chemotherapy toxicities in patients receiving anti-cancer treatments. Cancer Chemother Pharmacol. 2018;82(1):1-29. doi:10.1007/s00280-018-3614-8 29876640

[18] Stene GB, Helbostad JL, Amundsen T, Changes in skeletal muscle mass during palliative chemotherapy in patients with advanced lung cancer. Acta Oncol. 2015;54(3):340-348. doi:10.3109/0284186X.2014.953259 25225010

[19] Prado CM, Bekaii-Saab T, Doyle LA, Skeletal muscle anabolism is a side effect of therapy with the MEK inhibitor: selumetinib in patients with cholangiocarcinoma. Br J Cancer. 2012;106(10):1583-1586. doi:10.1038/bjc.2012.144 22510747 PMC3349178

[20] Blauwhoff-Buskermolen S, Versteeg KS, de van der Schueren MA, Loss of muscle mass during chemotherapy is predictive for poor survival of patients with metastatic colorectal cancer. J Clin Oncol. 2016;34(12):1339-1344. doi:10.1200/JCO.2015.63.6043 26903572

[21] Wildes TM Make time for gait speed: vital to staging the aging. Blood. 2019;134(4):334-336. doi:10.1182/blood.2019001335 31345925

[22] Liu MA, DuMontier C, Murillo A, Gait speed, grip strength, and clinical outcomes in older patients with hematologic malignancies. Blood. 2019;134(4):374-382. doi:10.1182/blood.2019000758 31167800 PMC6659254

[23] Klepin HD, Geiger AM, Tooze JA, ; Health, Aging and Body Composition Study Physical performance and subsequent disability and survival in older adults with malignancy: results from the health, aging and body composition study. J Am Geriatr Soc. 2010;58(1):76-82. doi:10.1111/j.1532-5415.2009.02620.x 20122042 PMC3760384

[24] Shachar SS, Williams GR, Muss HB, Nishijima TF Prognostic value of sarcopenia in adults with solid tumours: a meta-analysis and systematic review. Eur J Cancer. 2016;57:58-67. doi:10.1016/j.ejca.2015.12.030 26882087

[25] Goodpaster BH, Park SW, Harris TB, The loss of skeletal muscle strength, mass, and quality in older adults: the health, aging and body composition study. J Gerontol A Biol Sci Med Sci. 2006;61(10):1059-1064. doi:10.1093/gerona/61.10.1059 17077199

[26] Chen L, Nelson DR, Zhao Y, Cui Z, Johnston JA Relationship between muscle mass and muscle strength, and the impact of comorbidities: a population-based, cross-sectional study of older adults in the United States. BMC Geriatr. 2013;13:74. doi:10.1186/1471-2318-13-74 23865675 PMC3765109

[27] Rolland YM, Perry HM III, Patrick P, Banks WA, Morley JE Loss of appendicular muscle mass and loss of muscle strength in young postmenopausal women. J Gerontol A Biol Sci Med Sci. 2007;62(3):330-335. doi:10.1093/gerona/62.3.330 17389732

[28] Williams GR, Deal AM, Muss HB, Skeletal muscle measures and physical function in older adults with cancer: sarcopenia or myopenia? Oncotarget. 2017;8(20):33658-33665. doi:10.18632/oncotarget.16866 28431396 PMC5464899

[29] Goodpaster BH, Carlson CL, Visser M, Attenuation of skeletal muscle and strength in the elderly: the Health ABC Study. J Appl Physiol (1985). 2001;90(6):2157-2165. doi:10.1152/jappl.2001.90.6.2157 11356778

[30] Dunne RF, Loh KP, Williams GR, Jatoi A, Mustian KM, Mohile SG Cachexia and sarcopenia in older adults with cancer: a comprehensive review. Cancers (Basel). 2019;11(12):E1861. doi:10.3390/cancers11121861 31769421 PMC6966439

[31] Rolland Y, Abellan van Kan G, Gillette-Guyonnet S, Vellas B Cachexia versus sarcopenia. Curr Opin Clin Nutr Metab Care. 2011;14(1):15-21. doi:10.1097/MCO.0b013e328340c2c2 21076295

[32] Abramowitz MK, Hall CB, Amodu A, Sharma D, Androga L, Hawkins M Muscle mass, BMI, and mortality among adults in the United States: a population-based cohort study. PLoS One. 2018;13(4):e0194697. doi:10.1371/journal.pone.0194697 29641540 PMC5894968

